# The first fossil immature of Elmidae: an unusual riffle beetle larva preserved in Baltic amber

**DOI:** 10.7717/peerj.13025

**Published:** 2022-04-07

**Authors:** Ana Zippel, Viktor A. Baranov, Jörg U. Hammel, Marie K. Hörnig, Carolin Haug, Joachim T. Haug

**Affiliations:** 1 Ludwig-Maximilians-University of Munich, Biocenter, Munich, Germany; 2 Institute of Materials Physics, Helmholtz-Zentrum Hereon, Geesthacht, Germany; 3 University of Greifswald, Zoological Institute and Museum, Cytology and Evolutionary Biology, Greifswald, Germany; 4 GeoBio-Center at LMU, Munich, Germany

**Keywords:** Elmidae, Eocene, Amber, Processes, Operculum

## Abstract

Elmidae, riffle beetles, have both adult and immature stages that show specializations for water environments. Fossils of adults of Elmidae are already known from amber, however a record of immatures was so far lacking. We report here the first fossil larva of Elmidae, preserved in Baltic amber. To be able to access details of the body hidden by inclusions and “Verlumung” we conducted, in addition to optical documentation methods, micro-CT and synchrotron documentation methods. The larva is characterised by prominent dorso-lateral and lateral processes and a plate-like ventral operculum at the end of the abdomen. The new fossil has similarities in the general body shape and the prominent characters with some modern larvae of Elmidae. The posterior protrusions on the trunk end possibly represent gills, which would imply that fossil larvae of Elmidae also led a water-related life style similar to modern representatives.

## Introduction

Freshwater is a major type of habitat in modern-day ecosystems and was so in the past, at least from the Devonian onwards (about 400 million years ago) (Gueriau et al., 2016). Many may immediately think of crustacean ingroups as major components of freshwater faunas, such as representatives of Copepoda or Cladocera, or also crayfish (Astacida) or crabs (Brachyura). However, a much larger biomass is represented by another group of crustaceans (that often is still not recognised as such): Insecta. Especially the myriads of aquatic larvae (for challenges of this term in this aspect see Haug, 2020) of dragonflies, damselflies (both Odonata), mayflies (Ephemeroptera), stoneflies (Plecoptera), flies and midges (both Diptera), and many more represent an enormous share of the modern freshwater ecosystem. These representatives with at least one aquatic life stage are also known as “merolimnic” (Merritt & Cummins, 1996).

Among these aquatic forms are also many different types of beetles. This group entered freshwater habitats many times independently, leading to several diversification events (Wootton, 1988). In the extant freshwater fauna, beetles are making up 14% of all formally described species of Insecta (Sæther, 1993). In many cases, larvae and adults are aquatic, but nevertheless these differ strongly in their overall morphology, and coupled to that also in details of their ecology.

One of these merolimnic beetle groups is that of Elmidae, the riffle beetles. The group has a worldwide distribution with more than 1,300 formally described species. Some adults seem to be rather short-lived, surviving only for three weeks, as in the ingroup Larainae (Kodada & Jäch, 2005a p. 471), others seem quite long-lived with animals surviving up to ten years (Kodada & Jäch, 2005a). All adults are associated with running waters, and most of them indeed live within the water. All larvae are strictly aquatic.

In certain running waters, representatives of Elmidae may be the dominating animal life form (Kodada & Jäch, 2005a p. 484). In some areas of the world it seems they have even been consumed by humans (as a kind of seasoning) and therefore even had a commercial value (Philippi, 1864; Kodada & Jäch, 2005a p. 484). In a more modern function, representatives of Elmidae can be used for monitoring water quality (Hilsenhoff, 1982; Grasser, 1994; Moog & Jäch, 1995; Braukmann, 1997; Garcia-Criado, Fernandez-Alaez & Fernandez-Alaez, 1999; Garcia-Criado & Fernandez-Alaez, 2001). Adults and larvae seem to mostly feed on algae, which are scraped off from surfaces, in some cases also encrusting animals such as bryozoans are consumed. Few larvae seem also to feed on wood (LeSage & Harper, 1976), representing true wood-borers (Valente-Neto & Fonseca-Gessner, 2011), but still being aquatic. Larvae show a considerable variation from elongate cylindrical bodies to flattened, more or less onisciform ones.

While representatives of Elmidae seem to play an important role in modern freshwater ecosystems, the fossil record of the group is quite scarce (Peris et al., 2015). The oldest possible record is a specimen in Cretaceous amber from Spain (ca. 105 million years ago; Peris et al., 2015); yet the identity of this fossil as a representative of Elmidae has been doubted (Bukejs, Alekseev & Jäch, 2015). A younger fossil of Elmidae was reported from Myanmar amber (ca. 99 million years ago; Cai, Maier & Huang, 2018). In the younger Eocene Baltic amber (ca. 37 million years ago or less, Sadowski et al., 2017; Sadowski, Schmidt & Denk, 2020) two additional species have been reported (Wichard, Gröhn & Seredszus, 2009; Bukejs, Alekseev & Jäch, 2015).

Here we report an unusual beetle larva in Eocene Baltic amber. Its morphology was studied with optical, X-ray micro-CT and synchrotron micro-CT documentation methods. Based on comparison with extant forms we conclude that the larva is a fossil representative of Elmidae, making this a first record of a fossil larva of this group.We further discuss implications of this find.

## Material and Methods

### Material

The specimen is included in ca. 40 million-year-old (most likely 37 million-year-old; Priabonian) Eocene Baltic amber (Sadowski et al., 2017; Sadowski, Schmidt & Denk, 2020), PED 1414, deposited in the Palaeo-Evo-Devo Research Group Collection of Arthropods at the Ludwig-Maximilians-Universität München. The amber piece is approximately 20 mm long and 9.5 mm wide, containing a single larva of the group Insecta. Additionally, there are multiple detritus inclusions, small bubbles and grazes. Part of the larva is concealed with a white film (also known as “Verlumung”). The specimen was documented using optical, X-ray micro-computed tomography (µCT), and synchrotron radiation micro-computed tomography (SR-µCT) imaging.

### Optical documentation methods

Originally, the specimen was examined under a Keyence VHX-6000 digital microscope under different light settings (Haug, Müller & Sombke, 2013a; Hörnig et al., 2016). Stacks of photographs with differing focus levels were recorded from the specimen in different aspects, subsequently, these stacks were merged into single-focused images. Hence, all photographs are composite images. In cases when the structures were too large to fit into a single field of view, we have merged the adjacent photos into a panorama. We have also used HDR function to prevent areas that were excessively dark and too bright to appear on the photographs (cf. Haug et al., 2013b). All processing was done automatically by the built-in Keyence software.

### Micro-CT documentation methods

X-ray micro-computed tomography (µCT) was performed at the Imaging Center of the Department of Biology, University of Greifswald, using a XRadia XCT-200 (Carl Zeiss Microscopy GmbH, Jena, Germany). X-ray source settings were 40 kV and 8 W with a source-to-sample distance of 30 mm, sample-to-detector distance of 120 mm. The XRadia XCT-200 is equipped with switchable scintillator-objective lens units, in this case the 0.39×objective was used. 1600 projections with 1.25 s exposure time were recorded with binning 2, resulting in images of 1024 × 1024 px, with a system-based calculated pixel size of 13.49 µm. Tomographic reconstruction was performed (binning 1) using XMReconstructor (Carl Zeiss Microscopy GmbH, Jena, Germany), resulting in image stacks (TIFF format). Tiff image stacks were further post-processed in Fiji (Schindelin et al., 2012).

### Synchrotron documentation methods

Synchrotron radiation based X-ray micro-CT (SR-µCT) was performed at the PETRA III storage ring (DESY, Hamburg, Germany). The amber specimens were imaged with a photon energy of 18 keV and a sample-to-detector distance of 30 mm at the imaging beamline P05, operated by the Helmholtz-Zentrum Hereon (Greving et al., 2014; Wilde et al., 2016). The final reconstructed volume resulted in an effective pixel size of 2.56 µm. In the course of tomographic reconstruction a transport of intensity phase retrieval was applied. The beamline specific reconstruction was implemented in Matlab (The Math-Works) as described in Moosmann et al. (2014) using the Astra Toolbox (Van Aarle et al., 2015; Van Aarle et al., 2016).

### Visualisation of µCT and SR-µCT data

Volume renderings were conducted with Drishti ver. 2.6.6 (Limaye, 2012). To reduce the strain on the RAM and video card memory of the PC used, we downsized all of the original TIFF-stacks to 0.5 of the original size using Fiji ‘scale’ functionality (Schindelin et al., 2012); subsequently, we rendered the stacks in Drishti ver. 2.6.6 (Limaye, 2012). Surface mesh-model of the volume was constructed based on the downsized TIFF-stack in Drishti Paint v.2.6.4. Further volume renderings were performed in OSIRIX.

The volume renderings based on µCT and SR-µCT data of the fossil specimen were further processed with Adobe Photoshop CS2. Segmental units and appendages of the larva were marked in various colour nuances, which allows the reader to better follow our interpretation of the fossil.

### Comments on the used methods

Due to the strong “Verlumung” of the specimen, several details of the morphology are not or are only barely visible in the microscopic images. Therefore, µCT was used (see discussion in Hörnig et al., 2016) for an overview scan of the specimen. However, the contrast in µCT data turned out rather low for this specimen (contrast of amber inclusion is highly dependent on preservational conditions). Therefore, to record more details (*e.g.*, of the head region) SR-µCT was used in addition, which allowed us to record the specimen in enhanced contrast (phase contrast) with high resolution.

## Results

### Description of specimen PED 1414

#### General

**Figure 1:**
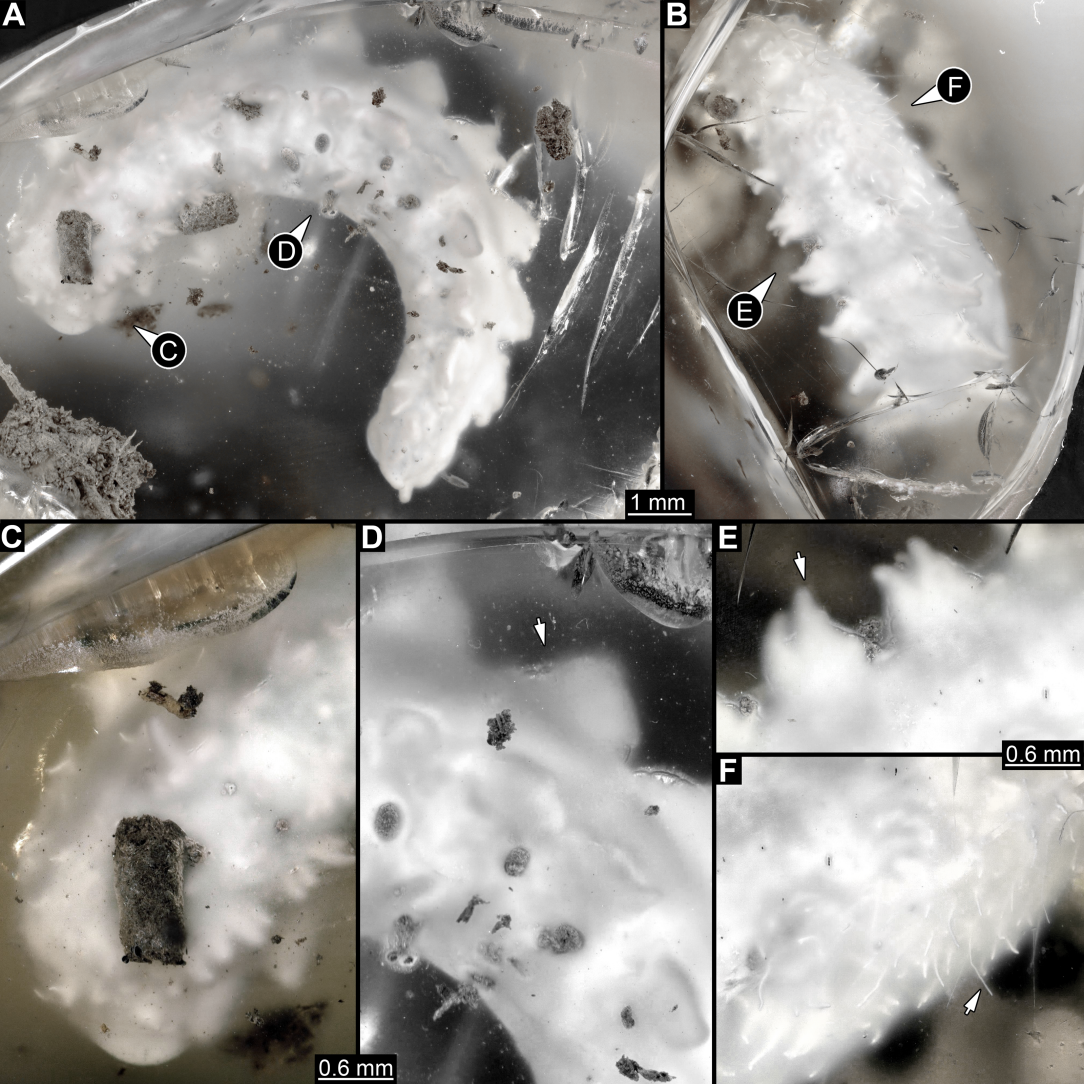
Composite digital images of larva of Elmidae in Eocene Baltic amber, PED 1414. (A) Lateral view. (B) Dorso-lateral view. (C–D) Close-ups. (C) Dorsal view, fringed distal ends of lateral processes discernible. (D) Dorso-lateral view, plate-like dorso-lateral processes well discernible (arrow). (E) Dorso-lateral process with fringed distal end (arrow). (F) Lateral view, body surface covered with white setae (single seta marked with arrow).

**Figure 2:**
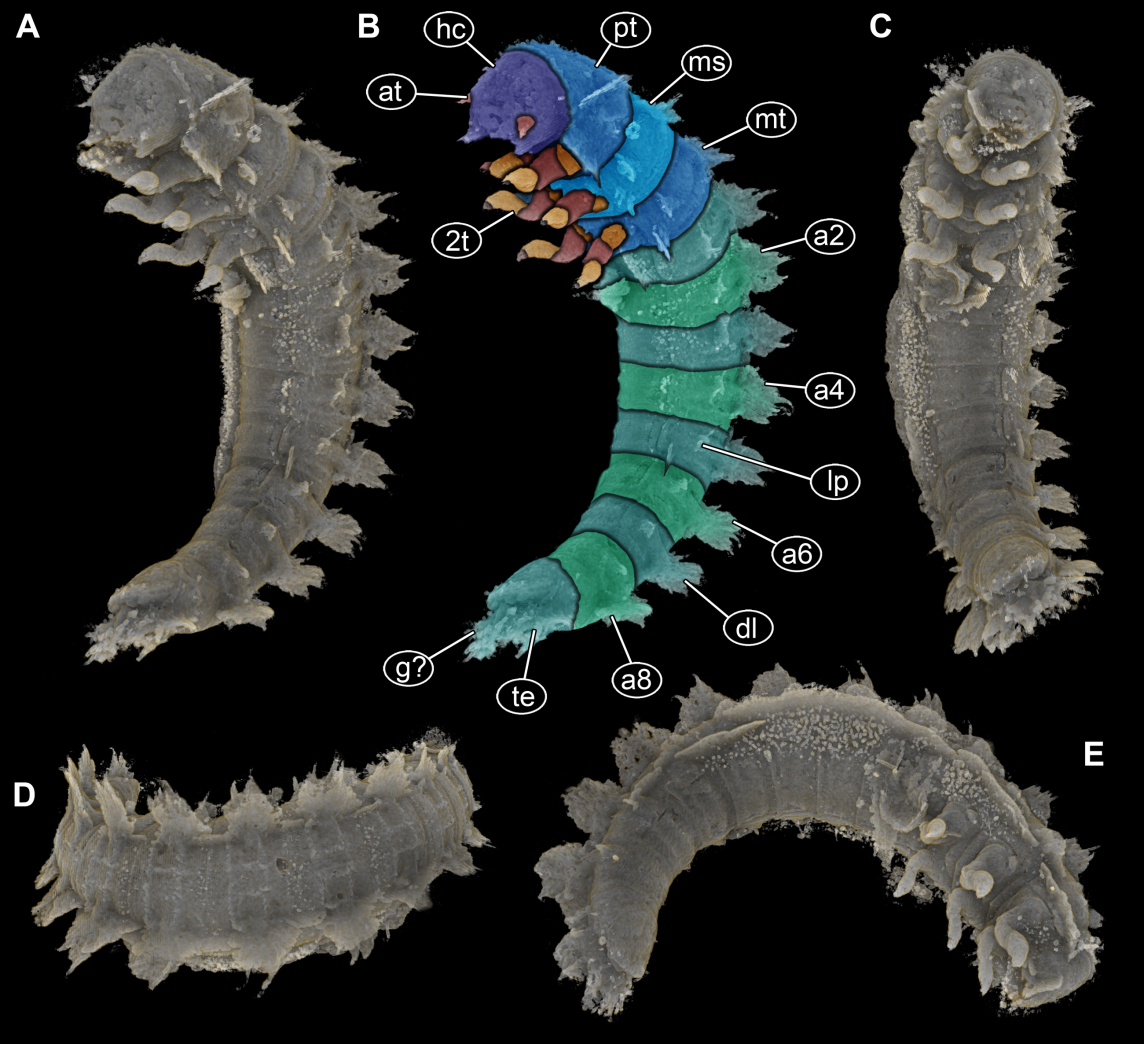
Volume renderings of PED 1414 based on micro-computed tomography (µCT). (A) Ventro-lateral view, dorsal processes in two rows on one side well discernible. (B) Color-marked version of (A). (C) Ventral view, thin structures protruding between operculum and dorsal region of trunk end well discernible. (D) Dorsal view, cylindrical curved body with two rows of processes on each side discernible. (E) Lateral view, strong mandibles discernible. Abbreviations: a2–8 = abdomen segments 2–8; at = antenna; dl = dorso-lateral process; g? = gills; hc = head capsule; lp = lateral process; ms = mesothorax; mt = metathorax; pt = prothorax; te = terminal end; 2t = second thorax segment appendages.

**Figure 3:**
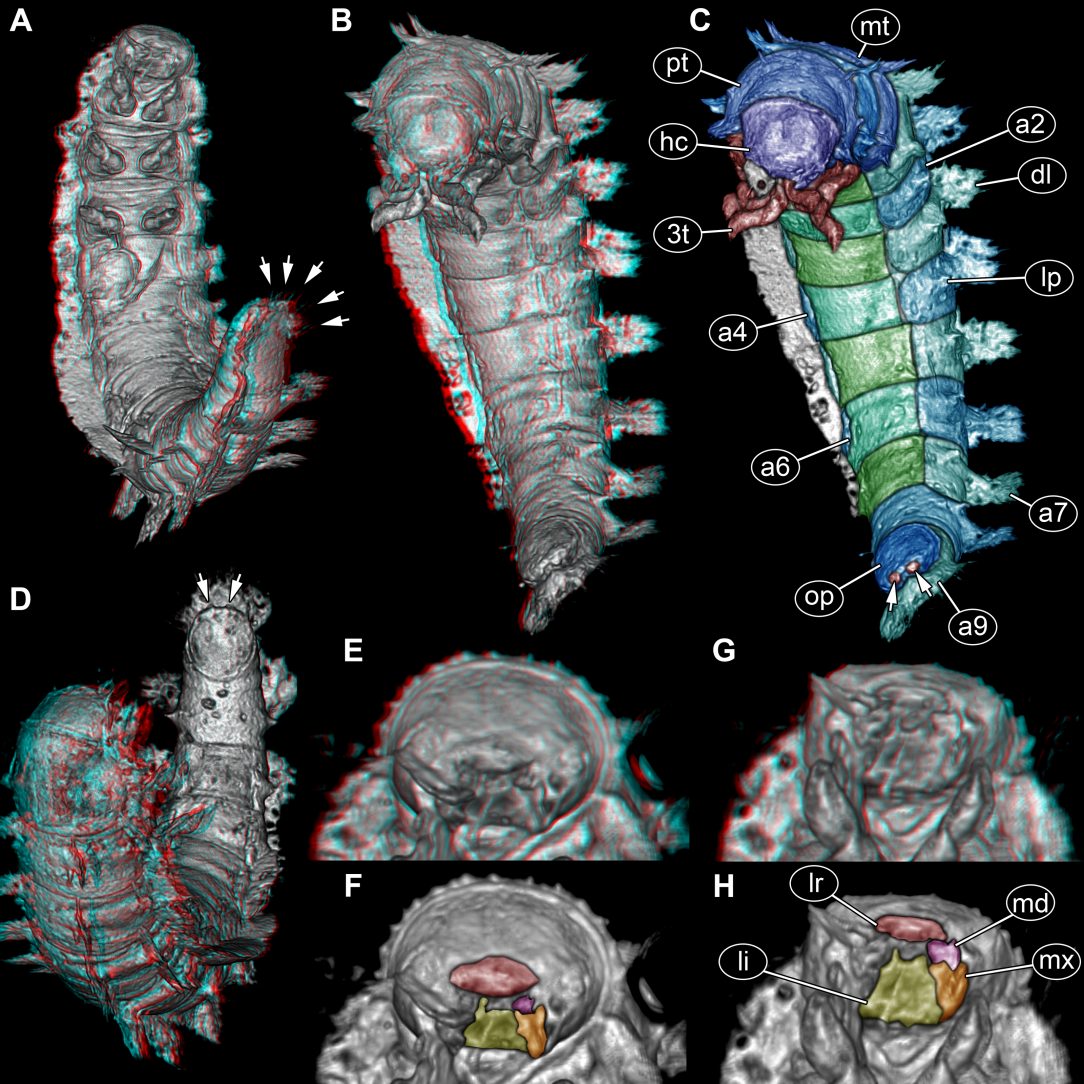
Volume renderings of fossil larva of Elmidae based on SR-µCT. (A) Ventral view of habitus, with abdominal terminal end in dorso-lateral view, possible posterior gills discernible (arrows). (B) Head and thorax in dorso-lateral view and abdomen in ventro-lateral view accessible. (C) Color-marked version of (B), terminal structures of operculum discernible (arrows). (D) Head and anterior part of trunk in dorsal view, posterior trunk in ventral view accessible, operculum with two terminal structures discernible (arrows). (E) Head with mouth parts in anterior view. (F) Color-marked version of (E). (G) Head with mouth parts in ventral view. (H) Color-marked version of (G). (A), (B), (D), (E), and (G) stereo anaglyphs, use red-cyan glasses to view. Abbreviations: a2–9 = abdomen segments 2–9; dl = dorso-lateral process; hc = head capsule; li = labium; lp = lateral process; lr = labrum; md = mandible; mt = metathorax; mx = maxilla; op = operculum; pt = prothorax; 3t = third thorax segment appendages.

Roughly cylindrical, slightly tapering body (Figs. 1A, 2C, 3B). Total body length approximately 13 mm. Body differentiated into an anterior region composed of head and a posterior region composed of trunk. Trunk differentiated into anterior trunk region (thorax) and a posterior trunk region (abdomen). Head prognathous (mouth parts facing forwards) (Figs. 2A, 3A).

#### Head

Head capsule trapezoid in dorsal view, widest posteriorly, slightly longer than maximum width, 1.1×(∼0.95 mm long and ∼0.85 mm wide) (Fig. 3C). Head capsule formed by ocular segment and post-ocular segments 1–5. Ocular segment with no discernible stemmata. Labrum (derivative of ocular segment) anteriorly narrowing, trapezoidal in ventral view (Figs. 3E–3H).

Antenna (appendage of post-ocular segment 1) positioned antero-laterally, with at least two elements. Second element bears structure at its distal end, not discernible if third element, sensorial structure (sensorium conical) or seta (Figs. 2A, 2B). No discernible structures of post-ocular segment 2 (intercalary segment).

Mandibles (paired appendages of post-ocular segment 3) partially hidden by other mouth parts, discernible part of triangular shape from anterior view (Figs. 3E, 3F). Only one mandible discernible, second presumed (Figs. 3E–3H).

Maxillae (paired appendages of post-ocular segment 4) proximally trapezoid in posterior view, distally with possible palp (Figs. 3E*,*3F). Only one maxilla discernible, second presumed (Figs. 3E*–*3H).

Labium (conjoined appendages of post-ocular segment 5) proximally symmetric trapezoid in posterior (functional ventral) view (Figs. 3E, 3F), distally with one discernible palp (second palp not discernible, but presumed) (Figs. 3E–3H).

#### Trunk

Prothorax sub-cylindrical in dorsal view (Fig. 3D), also longest segment of thorax, shorter than wide, 1.1×(∼1.2 mm long and ∼1.35 wide). With median longitudinal line optically dividing prothorax into two symmetrical parts. Meso- and metathorax sub-similar in shape (Fig. 3D), rectangular in dorsal view, wider than long, 1.5×. Mesothorax 1 mm long, metathorax slightly longer than mesothorax (∼1.1 mm long). Longitudinal line discernible on both, meso- and metathorax, but not as prominent as on prothorax.

All thorax segments with a pair of appendages (legs). Appendages approximately 2 mm long, each with five discernible elements: coxa, trochanter, femur, tibia, and distal claw (Figs. 2B, 2C, 2E).

Abdomen with nine units, anterior ones true segments; abdomen segments 1–8 sub-similar, approximately 1 mm long, width of segments gradually decreasing from anterior to posterior (1.6–1 mm) (Figs. 2C, 2D, 3B, 3C). Unit 9 trunk end (compound of several segments) longer, 1.5×(∼1.5 mm long), and narrower (∼0.9 mm wide) than rest of abdomen segments. In ventral view with prominent ventral plate-like structure (operculum) (Figs. 3C, 3D). Operculum oval-shaped in ventral view, longer than wide, 1.2×(∼0.9 mm long), with paired small protrusions at posterior end (Figs. 3C, 3D). At the posterior part of terminal end, thin structures protruding between operculum and dorsal region of trunk end (possible parts of gills) (Figs. 2B, 3A).

All trunk segments with two rows of plate-like lateral and dorso-lateral processes (Figs. 1A, 1D, 2B, 3C). Dorso-lateral processes with maximal length of ∼0.8 mm and proximal width between 0.6–1.2 mm, prominently larger than lateral processes (∼0.4 mm long and ∼0.3 mm proximally wide). Rims of the processes slightly serrated (Figs. 1B–1C, 1E). Lateral body surface bears setae (Figs. 1B, 1F).

## Discussion

### The new larva: a beetle

The distinct organisation of the body into head and elongate trunk, with the anterior three trunk segments bearing locomotory/ambulatory-type appendages (legs) is a strong identifier for the group Insecta (Figs. 2B, 3C). The absence of wings is either indicative of a position outside Pterygota or of an immature stage.

The number of segments in the posterior trunk (Figs. 2B, 3C) makes an interpretation as a non-pterygotan unlikely. Also the absence of prominent appendages on the abdomen makes a position in many ingroups of Pterygota with prominent appendages on the abdomen (*e.g.*, cerci in Polyneoptera) unlikely. Although mouth parts are incompletely preserved, it is clear that there is no beak as in condylognathans (Figs. 3E–3H). This leaves an interpretation as an immature (larva) of Holometabola as a likely one. Also here, the absence of appendages on the abdomen (Figs. 1A, 2B, 3C) immediately makes most groups unlikely (Lepidoptera, Mecoptera, Hymenoptera). The overall leg morphology (Fig. 2B) and mouth parts (Figs. 3F, 3H) are only compatible with an interpretation as a beetle larva.

### Beetle larvae with dorso-lateral and lateral processes

The most prominent feature of the here reported larva is the presence of prominent dorso-lateral (Fig. 1D) and lateral processes (Figs. 2B, 3C), reminiscent to that of the famous Japanese Kaiju Godzilla (Kalat, 2017). Lateral processes are quite common in different beetle larvae, as for example they are known in larvae of Micropeplidae (Newton, 1991 fig. 34.168 p. 335), Hydrophilidae (Spangler, 1991 fig. 34.297 p. 356), Chrysomelidae (Lawson, 1991 fig. 34.802a, 34.803a, 34.804a), or Brachypsectridae (recent review of all known larvae in Haug et al., 2021a).

Lateral and dorso-lateral processes are known in certain larvae of Coccinelidae (Lesage, 1991 fig. 34.570, 34571 p. 489), Lampyridae, Lycidae (Mjöberg, 1925 fig. 1, 2, 7 pl. III; Brues, 1941 pl. II p. 31, pl. III p. 32 upper; Levkanicova & Bocak, 2009 fig. 2 p. 215; Masek & Bocak, 2014 fig. 2, 3 p. 37, fig. 32, 33 p. 47, fig. 35, 36, 38, 39, 42 p. 49; Masek et al., 2014 fig. 12, 15, 16 p. 138; Kusy et al., 2019 fig. 2E p. 913), or Drilini (Baalbergen, Schelfhorst & Schilthuizen, 2016 fig. 3K p. 167). Still, in detail, most of these processes differ from those of the new fossil. Most processes are more spine-like or rod-like, while they are clearly plate-like in this fossil.

### Identity of the specimen

Besides the apparent processes on the back, the most prominent feature of the new larva is the posterior ventral plate-like structure (Figs. 3C, 3D). This distinct plate strongly resembles the operculum in certain polyphagan byrrhoidean beetle larvae, such as larvae of Elmidae, Dryopidae (Kodada & Jäch, 2005b fig. 18.3.3.H p. 504), Lutrochidae (Ide, Costa & Vanin, 2016 fig. 18.4 A p. 510), Chelonariidae (Beutel & Leschen, 2016 fig. 18.10.2B p. 545), as well as larvae of the myxophagan group Hydroscaphidae.

Smaller details are not fully resolved in the scans or tomographic data of the fossil larva, yet it appears that there is a pair of small terminal structures on the operculum in the fossil (marked with arrows in Figs. 3C, 3D). In extant larvae, there are small hooks in this region. According to Lawrence et al. (2011 p. 62) such hooks occur in larvae of Hydroscaphidae, Lutrochidae and Elmidae, but should be absent in Dryopidae and Chelonariidae. Even though there are examples of larvae of Dryopidae without hooks (Lawrence et al., 2011 fig. 83D p. 164), there are also larvae of Dryopidae with clearly present hooks (Kodada & Jäch, 2005b fig. 18.3.3.H p. 504).

**Figure 4:**
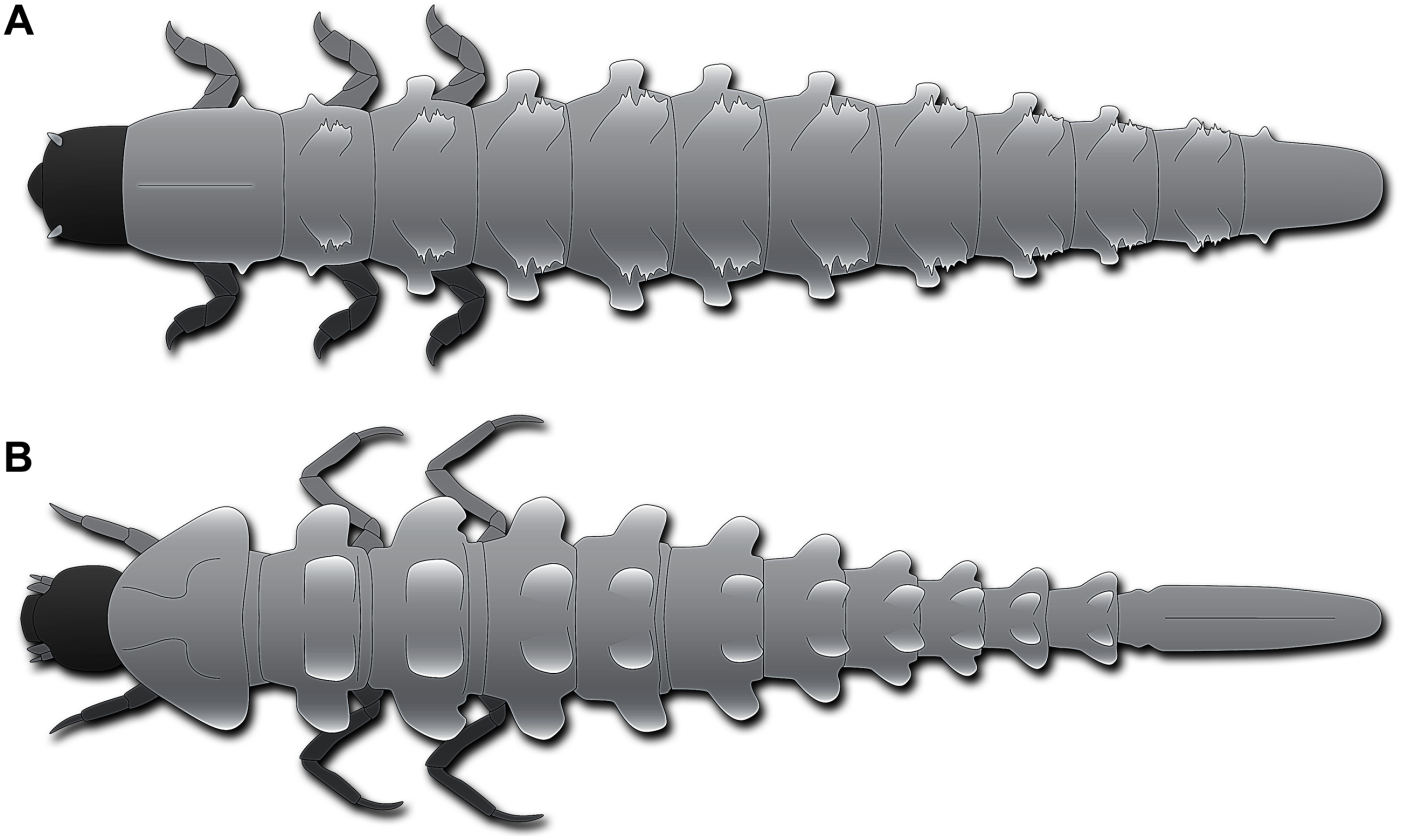
Habitus of fossil larva in comparison to modern one, both in dorsal view. (A) Schematic restoration of new larva. (B) Larva of *Neolimnius* (based on Shepard, Clavier & Cerdan, 2020 fig. 5 p. 4).

The roughly cylindrical, but slightly tapering body form (Figs. 1A, 2C, 2D, 3B, 3C, 4A) of the fossil larva is within the overall range of modern larvae. Many modern larvae are cylindrical, such as certain larvae of Elmidae, larvae of Dryopidae (Kodada & Jäch, 2005b fig. 18.3.4.A, B p. 505), Lutrochidae (Ide, Costa & Vanin, 2016 fig. 18.4 A p. 510), and Chelonariidae (Beutel & Leschen, 2016 fig. 18.10.2A, B p. 545). Other larvae of Elmidae and those of Hydroscaphidae are stronger fusiform (Vanin, Beutel & Arce-Perez, 2016), *i.e.,* strongly tapering towards the posterior.

Most of the modern-day byrrhoidean larvae and those of Hydroscaphidae lack any processes. However, there are indeed certain larvae of Elmidae that possess plate-like processes on the trunk segments dorso-laterally and laterally. Most prominently, such processes are developed in *Neolimnius* (Shepard, Clavier & Cerdan, 2020 fig. 5 p. 4), less so in *Austrolimnius* (Shepard, Clavier & Cerdan, 2020 fig. 4 p. 4). Although accessible details are limited, it appears that the rims of the processes are slightly serrated. In larvae of *Neolimnius* (Shepard, Clavier & Cerdan, 2020 fig. 5 p. 4) the dorso-lateral processes are quite comparable in relative size to those in the new fossil larva (Figs. 4A, 4B). Different from the fossil larva, dorso-lateral and lateral processes are more or less of the same size (Shepard, Clavier & Cerdan, 2020 fig. 4, 5 p. 4), while in the fossil the dorso-lateral processes are significantly larger than the lateral processes (Figs. 4A, 4B).

The modern larvae of Elmidae bearing processes differ in the morphology of the trunk end from that of the fossil. In the modern larvae, it is very elongate and slender (Fig. 4B) with the operculum being situated far posterior, in the posterior quarter of the trunk end. In the fossil larva, the trunk end is much shorter (Fig. 4A), the operculum far less posteriorly in the anterior half of the trunk end. This condition is much more similar to other larvae of Elmidae (Čiampor Jr & Ribera, 2006 fig. 40 p. 634; Shepard, Clavier & Cerdan, 2020 fig. 3 p. 4; fig. 7 p. 5, fig1. 4 p. 6). In other modern byrrhoidean larvae, the operculum is much further anterior, as in larvae of Dryopidae (Kodada & Jäch, 2005b fig. 18.3.3A, H p. 504, fig. 18.3.4A, B p. 505), Lutrochidae (Ide, Costa & Vanin, 2016 fig. 18.4 A p. 510), or Chelonariidae (Beutel & Leschen, 2016 fig. 18.10.2A, B p. 545).

An additional minor detail of the fossil interesting in this aspect are the thin structures protruding from between the operculum and the dorsal side of the trunk end (Figs. 2B, 3A). In modern larvae, such thin structures in this area represent the distal parts of the gills.

With all these details, we interpret the fossil larva as a representative of the group Elmidae. Although there is no modern larva with the exact same character combination as the fossil, all of these characters are seen in different modern larvae within the group. No observed detail of the fossil larva would argue against such an interpretation, however, the larva has a character combination not seen in modern larvae.

### The fossil record of the group Byrrhoidea

As already mentioned, there are only very few specimens of fossil representatives of Elmidae known (Bukejs, Alekseev & Jäch, 2015; Peris et al., 2015; Cai, Maier & Huang, 2018). Also the larger group, Byrrhoidea, seems in general known from very few fossils in amber (Pütz, Hernando & Ribera, 2004; Alekseev & Jäch, 2016; Hernando, Szawaryn & Ribera, 2018; Alekseev et al., 2021), but more common in other types of deposits (Kirejtshuk & Nel, 2016). The new fossil is an important addition to the amber record of the group, representing the first fossil larva of Elmidae.

It remains unclear whether the larva represents a new species. As two formally described species are already known in Baltic amber (Bukejs, Alekseev & Jäch, 2015) we cannot exclude that the larva is an immature stage of one of these two species.

### Life style of the new larva

All modern larvae of Elmidae are aquatic, living in running waters (except for the larvae of *Holcelmis* from Bolivia, which seems to live in stagnant water; Hinton, 1973; Merritt & Cummins, 1996). It seems therefore likely that this was the case also for the new fossil larva. The thin structures protruding between the operculum and the dorsal part of the trunk end (Figs. 2B, 3A) are likely to represent parts of gills supporting this interpretation. Most modern larvae feed by scraping off algae and other organisms from surfaces (Kodada & Jäch, 2005a), few bore into the wood while submerged in water (LeSage & Harper, 1976; Valente-Neto & Fonseca-Gessner, 2011). Even if it does not seem obvious, aquatic insects are astonishingly common in amber, especially adults but also immatures, and in fact far from only being caught in amber by chance (Wichard, Gröhn & Seredszus, 2009; Weitschat & Wichard, 2010; Horváth et al., 2021). There are several different scenarios suggested in the literature how larvae of aquatic insects might be enclosed in resin, such as (1) a resin flow into water-filled tree holes where aquatic insect larvae could be captured or (2) a resin flow from bark to the forest floor which reaches a water body (Schmidt & Dilcher, 2007), different scenarios summarised in Horváth et al. (2021), see also Schädel, Perrichot & Haug (2019 fig. 3). These scenarios require a close proximity of resin-producing trees and the water body where the enclosed animals lived in. Schmidt & Dilcher (2007) demonstrated that extant cypress forests, likely similar to some habitats in the Baltic amber forest, have abundant underwater resin flows. These resin flows are very efficient in trapping large and agile merolimnic insects, such as larvae of Elmidae. This is mostly due to the slow rate of polymerization of the resin flows and the size of the flows, reaching into dozens of centimetres (Schmidt & Dilcher, 2007; Wichard, Gröhn & Seredszus, 2009; Schädel, Perrichot & Haug, 2019; Schädel et al., 2021).

Peris & Rust (2020) recognised that most beetles preserved in amber are associated with wood, but that wood borers seem to be rare. Yet, this may be related to the fact that beetle larvae are much rarer reported than adults. Detailed search for such wood borer-type larvae reveals that they are indeed present in different ambers (Haug et al., 2021b; Zippel et al., 2022; Zippel et al., 2022b; A Zippel, C Haug, P Müller & JT Haug, 2022, unpublished data). This may be seen as an argument that the new specimen might have been somehow wood-associated, but that could mean as well that the larva was scraping off algae from wood. Unfortunately, we have no report on the exact life habits of modern-day larvae with dorso-lateral and lateral processes. If these are used for a specific function, we could assume a similar one for the fossil. Until more data on the extant larvae become available, this must remain unclear also for the fossil.

## Additional Information and Declarations

### Data Availability

The following information was supplied regarding data availability: The raw data will be available on MorphDBase: Haug, C. (2022): C_Haug_20220222-M-18.1.
https://www.morphdbase.de/?C_Haug_20220222-M-18.1.
